# Association between ultra-processed dietary pattern and bullying: the role of deviant behaviors

**DOI:** 10.3389/fnut.2024.1352258

**Published:** 2024-07-04

**Authors:** Letícia Martins Okada, Emanuele Souza Marques, Renata Bertazzi Levy, Vivian Siqueira Santos Gonçalves, Maria Fernanda Tourinho Peres, Catarina Machado Azeredo

**Affiliations:** ^1^Programa de Pós-Graduação em Ciências da Saúde, Faculdade de Medicina, Universidade Federal de Uberlândia, Uberlândia, Brazil; ^2^Instituto de Medicina Social Hesio Cordeiro, Universidade do Estado do Rio de Janeiro, Rio de Janeiro, Brazil; ^3^Departamento de Medicina Preventiva, Faculdade de Medicina, Universidade de São Paulo, São Paulo, Brazil; ^4^Programa de Pós-graduação em Saúde Coletiva, Faculdade de Ciências da Saúde, Universidade de Brasília, Brasília, Brazil

**Keywords:** ultra-processed food, dietary patterns, bullying, deviant behavior, mediation, adolescents

## Abstract

**Background:**

Ultra-processed foods have been associated with several negative outcomes, but it is not clear whether they are related to bullying perpetration. Moreover, no previous study has investigated the potential role of deviant behaviors as a mediator of this association. Our objective was to evaluate the association between ultra-processed dietary pattern and bullying, and the mediating effect of deviant behaviors in this association, among school adolescents.

**Methods:**

We used data from a representative sample of 9th grade Brazilian adolescents (*N* = 2,212) from the São Paulo Project for the social development of children and adolescents (SP-PROSO). Exploratory factor analysis was used to obtain the dietary patterns, through questions of frequency of consumption in the last week of several foods. The ultra-processed dietary pattern was considered as exposure. The outcomes were the types of bullying (any type, social exclusion, psychological/verbal aggression, physical aggression, property destruction, and sexual harassment). Deviant behaviors (mediator) were assessed through a score. Mediation analyses were carried out using logistic regression based on the KHB method.

**Results:**

After adjusting for covariates, the mediating effect of deviant behaviors was found in the association between ultra-processed dietary pattern and all the types of bullying perpetration, especially for psychological/verbal aggression (39.4%). A small mediating effect of deviant behaviors in the association of ultra-processed dietary pattern with physical aggression (17.7%) and property destruction (18.5%) was observed, but this effect explained only a small portion of the total effect of such association (significant direct effect).

**Conclusion:**

The ultra-processed dietary pattern was associated with bullying, and the association was mediated through deviant behaviors. Policies and actions for improving the adolescent’s diet and managing the adoption of deviant and bullying behaviors by this public are required.

## Graphical abstract

Graphical abstract of the possible mechanisms by which the ultra-processed dietary pattern is associated with the perpetration of bullying. This figure was created using BioRender.com.

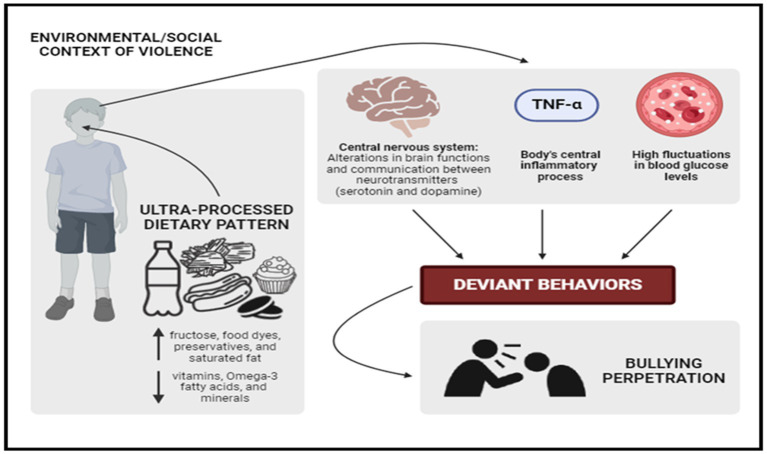

## 1 Introduction

Bullying has become a major public health problem around the world (1, 2) and is recognized as a subset of violent behavior that can be defined through three specific characteristics: intentionality, repetitiveness, and power imbalance (3). In Brazil, findings from PeNSE 2019 (Brazilian National School-Based Health Survey) indicated that 12% of adolescents aged 13 to 17 years reported having practiced bullying against their peers, with a higher percentage among boys (14.6%) than among girls (9.5%) (4).

In recent years, only a few studies have investigated the association between food consumption and violent behaviors (5, 6), especially bullying perpetration (7–9). Our latest research (10) found that adolescents who had an unhealthy dietary pattern were more likely to perpetrate overt (e.g., physical aggression) and covert (e.g., social exclusion) forms of bullying. The lack of investigation of this association is striking, considering the significant body of literature that joins nutritional elements to mental health (11, 12), and ties problem behavior in childhood to specific aspects of brain development (13).

For optimal brain function, it is necessary to consume a sufficient number of vitamins, minerals, and other nutrients such as folate, zinc, iron, magnesium, and polyunsaturated fatty acids (12), which are predominantly found *in natura* or minimally processed foods (14). In Brazil, especially among adolescents (15), the consumption of these foods has been gradually replaced by the consumption of ultra-processed foods (16), defined according to the NOVA food classification system as industry products, mostly or completely made from ingredients and containing little or no whole foods in its formulation (14). The physiological element of diet on bullying, in addition to social and environmental elements (17), proposes that an unhealthy diet may influence adolescent’s behaviors by affecting the brain’s structural development, brain neurotransmitters, and brain functions (18, 19), which would contribute to engagement in bullying perpetration.

Among adolescents, there is a strong interrelationship between interpersonal violence, bullying behaviors, and delinquency (20, 21), which are offensive acts of violation of major rules, social norms, and even the current judicial order (22). Research shows that these deviant acts – e.g., bragging, lying, truancy, stealing, running away, and fighting – may be the result of unhealthy eating (23, 24) through the same proposed physiological pathway (18, 19) and are committed at an earlier age by future perpetrators (25). This mechanism warrants the importance of investigating our hypothesis tha**t** deviant behaviors are a possible mediator of the association between food consumption and bullying in adolescence.

Although the consumption of ultra-processed foods is increasing in the Brazilian diet (16), especially during and after the COVID-19 pandemic lockdown (26, 27), and the association between unhealthy eating and bullying perpetration has been reported in the literature (7–10), no study to date has broadly evaluated the consumption of these foods associated with bullying. Furthermore, as deviant behavior is a characteristic of those who bully especially the overt form (28), its mediating effect on the association between an ultra-processed dietary pattern and bullying perpetration needs to be clarified. In that regard, this study intends to elucidate the role of deviant behaviors in this association and evaluate adolescents’ food consumption through the dietary pattern (29), understanding that it represents a wide combination of ultra-processed foods that are usually part of their eating habits. It is also relevant to assess different forms of bullying and possibly find different mediation percentages of deviant behaviors for each type of perpetration, associated with the ultra-processed dietary pattern.

Therefore, the present study aims to evaluate the association between dietary pattern, especially the ultra-processed pattern and bullying perpetration, and to explore the mediating effect of deviant behaviors on this association, among school adolescents.

## 2 Methods

### 2.1 Data collection and sampling

We used data from the São Paulo Project for the social development of children and adolescents – SP-PROSO (*Projeto São Paulo para o desenvolvimento social de crianças e adolescentes*), a cross-sectional study conducted from August to November 2017 with a representative sample of 9th grade students from public and private schools in São Paulo, Brazil (30).

The sampling strategy used stratification by school type (state public schools, municipal public schools, and private schools in São Paulo) and clustering by school class, considered as the primary sampling unit. Of the 156 schools selected, 128 were randomly selected to start data collection. The remaining schools were drawn as reserves if it was necessary to complete the sample. After refusals (26 private schools and 8 public schools) and school non-response (3 private schools), 119 were included in the final sample. Eligible adolescents were those who were present in the classroom on the day of data collection, whose parents did not disallow them from participating, and who did not present any serious difficulty that might avoid understanding questions or prevent from answering them anonymously.

Participants were invited to sign an Informed Consent Term and to answer a paper-pencil structured questionnaire, with questions about sociodemographic and familiar characteristics, school environment, violence, and students’ food consumption and physical activity. The questionnaire was based on models previously used in the longitudinal study Zurich Project on the Social Development of Children (Z-PROSO) and Montevideo Project for the Social Development of Children and Adolescents (M-PROSO, *Proyecto Montevideo para el desarrollo social de niños y adolescentes*), later translated to Portuguese.

**Figure 1:**
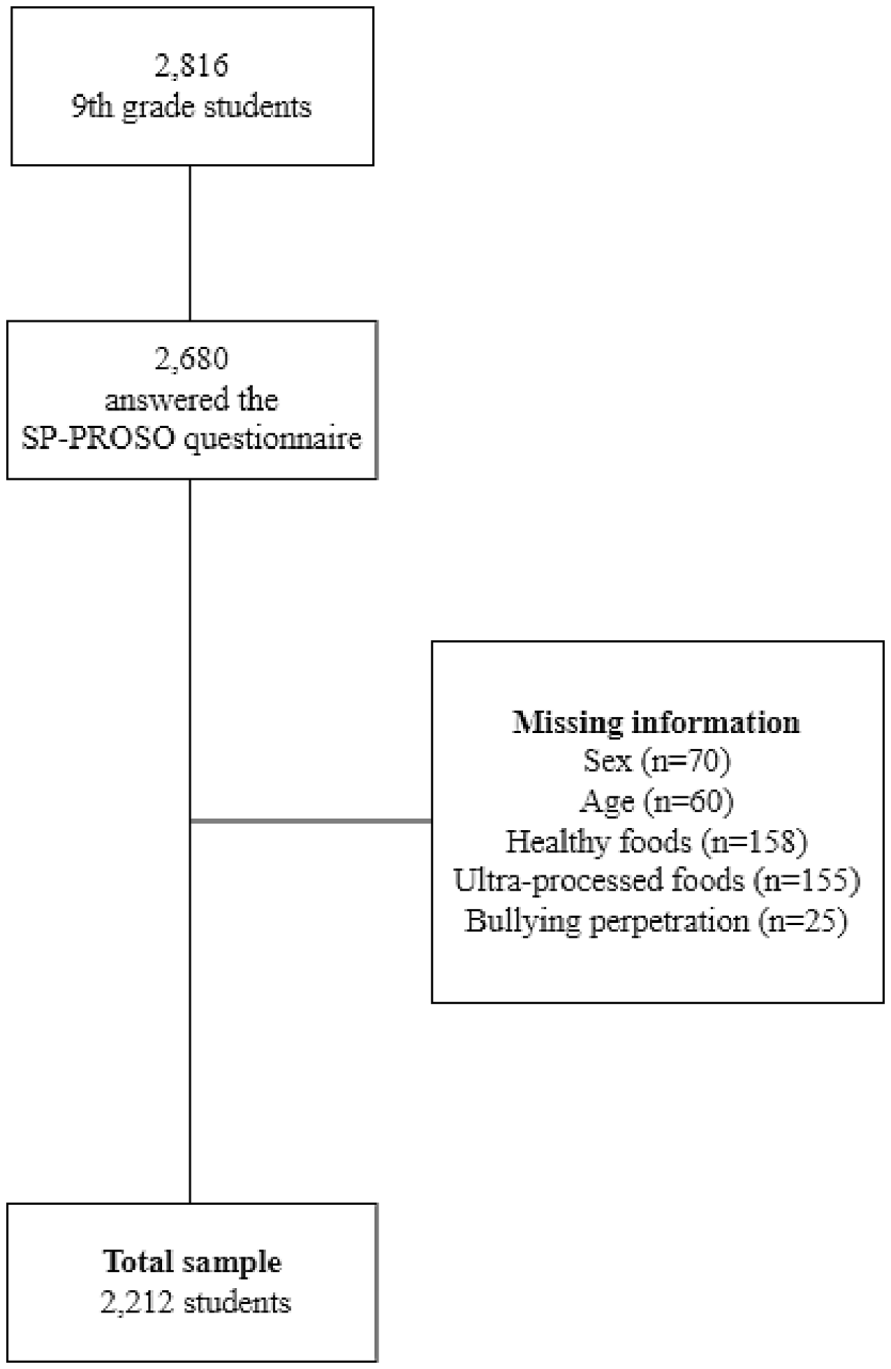
Flowchart of the total study sample (9th grade students), SP-PROSO.

Of the 2,816 ninth-grade students present on the survey day, 2,680 answered the questionnaire. The final sample size included in this study comprised 2,212 participants (Figure 1). More details of the sampling process are available in the SP-PROSO report (30).

### 2.2 Ultra-processed dietary pattern – exposure

The student’s food consumption was evaluated by the weekly consumption of healthy (beans, raw/cooked vegetables, and fruit/fruit salad) and ultra-processed foods (sausages, biscuits/cookies, package snacks/potato chips, candies, and sugary beverages). The frequencies were grouped in a dichotomous variable (<5 times/week or ≥ 5 times/week), hereinafter referred to as regular consumption (31). Subsequently, exploratory factor analysis was performed on the total sample to obtain the dietary patterns. This statistical method aggregates specific food items based on the degree to which they are correlated with one another in the data set. Orthogonal (varimax) rotation was applied for greater interpretability. The number of selected factors was chosen through the scree plot preview. Food items with a loading factor greater than 0.30 were considered in identifying the two dietary patterns (32). The “ultra-processed dietary pattern” was considered as the exposure in this study and the “healthy dietary pattern” as a covariate of the analyses. The sampling adequacy was assessed through the Kaiser-Meyer-Olkin (KMO) criteria, which takes values between 0 and 1, with small values generally meaning that variables have too little in common to justify a factor analysis. A value of KMO = 0.81 was obtained, meaning good adequacy (33).

### 2.3 Types of bullying perpetration – outcomes

In August 2017, the adolescents self-reported the frequency of bullying perpetration in the previous 12 months through five questions regarding specific types of perpetration, following the general question: “Since August 2016, how often have you (1) ignored or excluded on purpose, (2) made fun of or called names, (3) punched, kicked, bitten, or pulled the hair of, (4) taken, broken, or hidden on purpose some of the belongings of, and (5) sexually harassed other adolescent?” Possible answers were 1 = never; 2 = once or twice; 3 = from 3 to 10 times; 4 = once a month; 5 = once a week; 6 = every day. Bullying perpetration was considered when students reported having practiced this violence at least once a month in the last year (34). Each type of bullying perpetration (any type, social exclusion, psychological/verbal aggression, physical aggression, property destruction, and sexual harassment) was considered as an outcome of the present study.

### 2.4 Deviant behaviors – mediator

We have some arguments that scientifically support us to consider deviant behaviors as a possible mediator of the association between ultra-processed dietary pattern and bullying perpetration. In addition to the physiological pathway explored in the literature (19, 24, 25), we examined whether ultra-processed dietary pattern was associated with bullying after controlling for deviant acts. After the adjustment, the ultra-processed dietary pattern remained significantly associated only with physical aggression and sexual harassment (*p* < 0.05), which means that the association between exposure and the other types of bullying perpetration (social exclusion, psychological/verbal aggression, and property destruction) may occur via deviant behaviors.

We also performed interaction analysis to test whether deviant behaviors would modify associations between the ultra-processed dietary pattern and our outcomes. As the interaction term was statistically significant only for the association between ultra-processed dietary pattern and sexual harassment (*p* < 0.05), there is not enough evidence of effect modification by deviant behaviors, which leads us to test it as a mediator of our associations of interest.

Deviant behaviors were measured by the adolescent’s engagement or not in 19 different types of delinquent actsin the previous 12 months of the survey, adapted from Nivette et al. (35). Acts included skipping class on purpose, cheating on school test, running away from home, stealing at home, stealing at school, shoplifting something worth more than 150 reais, shoplifting something worth less than 150 reais, vehicle theft, driving without a license, illegally downloading files from the internet, burglary and stealing from a car, drug dealing, using the train, bus or subway without having paid the ticket, graffitiing, vandalism, carrying a weapon, threatening and extortion, robbery, and assault. We opted for a variety score because it is generally considered a more valid indicator of an individual’s involvement in deviant behavior (intensity of deviance) compared to frequency measures of the total number of deviant acts (36). The questions used in our study to construct the score reflect a variety of acts that are considered deviant across different populations within the context of adolescence (35, 37, 38), in addition to being considered valid self-report measures of delinquency (39, 40).

### 2.5 Covariates

We assessed sex (male, female), age (in years), skin color/race (white, non-white, non-response), maternal educational level (incomplete middle school, complete middle school, complete high school, complete higher education, non-response), physical fitness (<420 min/week, ≥420 min/week), screen-based sedentary activities (<3 h/day, ≥3 h/day, non-response), students’ perception of community violence (low, medium, high), school administrative status (private, public, and non-response), and healthy dietary pattern. These covariates may be considered confounding variables, which are associated with both the outcome variable (bullying perpetration) and the exposure variable (ultra-processed dietary pattern), and should be appropriately controlled in the statistical analyses (41).

### 2.6 Data analyses

**Figure 2:**
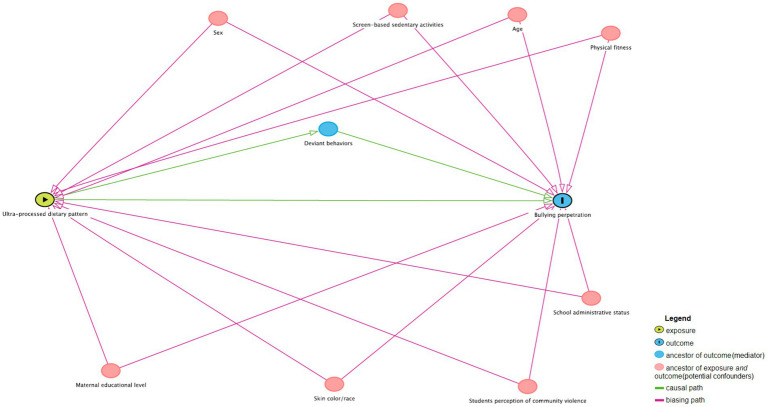
Directed acyclic graph (DAG) for the association between ultra-processed dietary pattern and bullying perpetration.

The analyses were performed taking into consideration the sample design and weights to represent the population of school adolescents from São Paulo. Figure 2 presents a directed acyclic graph (DAG) for the association between the ultra-processed dietary pattern and bullying perpetration, which was constructed using the DAGitty graphical interface (42). All covariates were also included in the DAG, which graphically represents the probable causal structure between the study variables, in addition to identifying those that must be controlled to avoid analytical errors that can lead to false effect estimates (43). First, a descriptive analysis of the total sample was performed to obtain the percentages and their respective confidence intervals (95%CI) of the variables of interest. Mean and standard deviation for deviant behaviors (mediation variable) were also obtained. Then, to assess the role of deviant behaviors (see Figure 2) in the association between ultra-processed dietary pattern and bullying perpetration, mediational analyses were performed using logistic regression based on the KHB method proposed by Breen et al. (44). It decomposes the total effect (the effect of ultra-processed dietary pattern on bullying perpetration without the mediation variable) into direct effect (the effect of ultra-processed dietary pattern on bullying perpetration when controlling for the mediation variable) and indirect (mediating) effect (the effect of ultra-processed dietary pattern on bullying perpetration through the mediation variable).

The KHB method also allows for the calculation of the proportion of the mediating effect, which is considered as the percentage of the main association that can be explained by the mediators. The mediational analyses were also controlled for all the above-mentioned covariates. Results were expressed as coefficients with their respective 95% confidence intervals. *p*-values <0.05 were considered statistically significant, and all statistical analyses were performed using Stata SE version 13.0 software (45).

## 3 Results

The study sample characteristics are summarized in Table 1. Adolescents showed a similar distribution regarding sex (52.6% male) and had an average age of 14.9 years (SD: 0.69). Regular consumption (≥5 times/week) of sugary beverages (46.1%) and biscuits/cookies (40.9%) were the most frequent among adolescents. Regarding bullying perpetration, 14.6% of students perpetrated any type of bullying, with psychological/verbal aggression being the most frequent type (10.1%). The mean and standard deviation of deviant acts committed by adolescents were also presented.

**Table 1:** Description of the 9th grade students’ sample (*n* = 2,680).

Variables	Total
*Sex*	
Male	52.6 (50.9–54.3)
Female	47.4 (45.7–49.1)
Age (mean)	14.88 (0.69)
*Race/color*	
White	43.8 (41.3–46.4)
Non-white	55.2 (52.7–57.8)
Non-response	0.9 (0.5–1.5)
*Maternal educational level*	
Incomplete middle school	12.4 (10.9–14.0)
Complete middle school	10.6 (9.4–12.0)
Complete high school	26.3 (23.6–29.3)
Complete higher education	24.8 (21.9–27.8)
Non-response	25.8 (23.7–28.1)
*Physical fitness*	
< 420 min/week	89.7 (87.5–91.5)
≥ 420 min/week	10.3 (8.4–12.5)
*Screen-based sedentary activities*	
< 3 h/day	34.5 (32.2–37.0)
≥ 3 h/day	62.3 (59.8–64.8)
Non-response	3.1 (2.2–4.3)
*Students’ perception of community violence*	
Low	36.8 (34.1–39.6)
Medium	33.1 (30.8–35.4)
High	30.1 (27.5–32.8)
*School administrative status*	
Private	30.2 (28.4–32.2)
Public	66.0 (61.0–70.6)
Non-response	3.7 (1.1–12.1)
*Food consumption^a^*	
Sausages	21.8 (19.6–24.2)
Biscuits/cookies	40.9 (38.9–43.0)
Package snacks/Potato chips	23.3 (21.3–25.4)
Candies	37.8 (35.5–40.0)
Sugary beverages	46.1 (43.6–48.7)
*Bullying perpetration*	
Any type	14.6 (12.8–16.6)
Social exclusion	4.4 (3.6–5.4)
Psychological/verbal aggression	10.1 (8.9–11.4)
Physical aggression	3.2 (2.4–4.2)
Property destruction	5.6 (4.5–7.0)
Sexual harassment	1.8 (1.3–2.6)
Deviant behaviors (mean)	2.44 (2.13)

SP-PROSO, São Paulo Project for the social development of children and adolescents. CI, confidence interval; SD, standard deviation. Data presented as % (95% CI) or mean (SD).

a Regular consumption: ≥ 5 times/week.

For the whole sample, two dietary patterns were retained, explaining 53.66% of the variance. The first pattern consisting of sausages, biscuits/cookies, package snacks/potato chips, candies, and sugary beverages was labeled “ultra-processed dietary pattern” – the study’s exposure. The other pattern which includes beans, raw/cooked vegetables, and fruit/fruit salad was labeled “healthy dietary pattern” (Table 2).

**Table 2:** Factor structure of food consumption among 9th grade students (*n* = 2,212).

Variables	Ultra-processed dietary pattern Factor 1	Healthy dietary pattern Factor 2
Beans	0.1855	**0.4999**
Raw/cooked vegetables	0.0481	**0.8366**
Fruit/fruit salad	0.2032	**0.7579**
Sausages	**0.6616**	0.1685
Biscuits/cookies	**0.7594**	0.1539
Package snacks/Potato chips	**0.7779**	0.1524
Candies	**0.7336**	0.0807
Sugary beverages	**0.6690**	0.0600
Eigenvalue	3.05	1.23
Explained variance (%)	33.54	20.12
Cumulative explained variance (%)	33.54	53.66

SP-PROSO, São Paulo Project for the social development of children and adolescents. Bold data reflect that the variable has loaded in the pattern.

The results from the mediation analyses are shown in Table 3. After adjusting for potential confounders, the mediating effect (indirect effect) of deviant behaviors in the association of ultra-processed dietary pattern with all types of bullying perpetration was observed, highlighting that 37.7% of the association between ultra-processed dietary pattern and perpetration of any type of bullying was mediated by deviant behaviors. A similar mediation percentage (39.4%) was found for psychological/verbal aggression. The mediation percentages for the perpetration of social exclusion and sexual harassment were 21.1 and 12.5%, respectively. Our findings also indicated that deviant behaviors had a positive effect on the perpetration of physical aggression and property destruction, but this effect explained only a small portion of the effect of ultra-processed dietary pattern on these types of bullying (17.7 and 18.5%, respectively); in contrast, the direct effect of ultra-processed dietary pattern on perpetration of physical aggression (82.3%) and property destruction (81.5%) was major.

**Table 3:** Logistic regression analyses of the association between ultra-processed dietary pattern and bullying perpetration (outcome) with deviant behaviors as mediator (KHB method) (*n* = 2,209).

	Bullying perpetration											
	Anytype		Social exclusion		Psychological/verbal aggression		Physical aggression		Property destruction		Sexual harassment	
Mediators	Coefficient	(95% CI)	Coefficient	(95% CI)	Coefficient	(95% CI)	Coefficient	(95% CI)	Coefficient	(95% CI)	Coefficient	(95% CI)
Total	**0.18**	**(0.04–0.32)**	**0.27**	**(0.01–0.52)**	0.14	(−0.02–0.31)	**0.49**	**(0.22–0.77)**	**0.29**	**(0.06–0.51)**	**0.50**	**(0.02–0.98)**
Direct	0.11	(−0.03–0.25)	0.21	(−0.04–0.46)	0.09	(−0.08–0.26)	**0.40**	**(0.13–0.68)**	**0.24**	**(0.01–0.46)**	0.44	(−0.03–0.91)
Indirect	**0.07**	**(0.03–0.10)**	**0.06**	**(0.02–0.09)**	**0.05**	**(0.03–0.09)**	**0.09**	**(0.04–0.13)**	**0.05**	**(0.02–0.08)**	**0.06**	**(0.02–0.10)**
Mediation percentage	**37.7%**	__	**21.1%**	__	**39.4%**	__	**17.7%**	__	**18.5%**	__	**12.5%**	__

SP-PROSO, São Paulo Project for the social development of children and adolescents. All models were adjusted for sex, age, race/color, maternal educational level, physical fitness, screen-based sedentary activities, students’ perception of community violence, school administrative status, and healthy dietary pattern. CI, confidence interval. In bold, significant associations (*p* < 0.05).

## 4 Discussion

This study found a mediating effect of deviant behaviors in the association of the ultra-processed dietary pattern with all types of bullying, with a high mediation percentage for psychological/verbal aggression (39.4%). Moreover, significant direct effects were found when analyzing the mediation of deviant behaviors in the association with physical aggression and property destruction.

Given that violence results from a complex causal process that considers the importance of the environmental/social context for the emergence of the violent act (17), there are two possible physiological mechanisms to explain the association between the ultra-processed dietary pattern and bullying perpetration represented by the illustration in the Graphical abstract. The first shows that unhealthy diets, such as the Western dietary pattern, could be associated with greater involvement in delinquent and/or aggressive behaviors (e.g., lying, stealing, arguing, sudden mood swings, and temper tantrums), and related to the perpetration of bullying (24). The Western diet is predominantly composed of foods rich in fructose, food dyes, and preservatives (46) and that lack essential nutrients such as vitamins, Omega-3 fatty acids, and minerals, denominated as ultra-processed foods (15). Fried foods (chicken and fish “nuggets”), commercially-baked goods (cakes, cookies, crackers), and packaged snacks (popcorn, potato chips, and candy) may lead to alterations in brain functions and communication between neurotransmitters like serotonin and dopamine (47, 48). Consequently, these alterations may be associated with an increase in deviant behavior, aggression, and violence perpetrated by adolescents (23, 49). Furthermore, a previous study found that a high-palmitic acid diet (similar to the Western diet fat composition) induced a central inflammatory process in the body (49) by increasing the secretion of TNF-ά (50, 51), which led to more anger and hostility (52) and, consequently, to the perpetration of bullying.

In the second mechanism proposed, daily consumption of soft drink components (e.g., aspartame) may trigger frequent nervousness and irritability (53) due to high fluctuations in blood glucose levels (54) caused by the functional deficit in serotonergic neurons in the central nervous system (55), which can lead to deviant behavior and perpetration of bullying (23). Some soft drinks (e.g., Coke) contain caffeine, which can be associated with conduct disorders and violent behavior among adolescents, due to several physiological actions in the central and peripheral nervous system (56).

According to our results, the deviant behaviors had a small positive mediating effect on the perpetration of physical aggression (17.7%) and property destruction (18.5%), which means that the association of the ultra-processed dietary pattern with these types of bullying (significant direct effect) may have occurred through mediators other than the deviant behaviors, which could not be tested in this study. To date, only one recent research (57) has evaluated the association between poor nutrition (e.g., high junk food intake) and bullying perpetration among deviant and non-deviant youths. The findings showed that bullying perpetration of non-deviant youth was more tightly influenced by high junk food intake than the bullying perpetration of deviant youth, due to their higher biological sensitivity to the effects of an unhealthy diet. According to the social push hypothesis (58), aggressive youths devoid of environmental risk – not involved in deviant behavior and without deviant peer affiliations – are often prone to be influenced by biosocial risk factors (such as nutritional inadequacies arising from unhealthy eating) that can “push” them towards aggressive acts, in relation to youths who are within a context of greater risk for deviant behavior. The idea is that the greater the intake of junk food, the less space available for the nutrients and chemicals needed to nourish the brain, which may lead to more violence (59). The literature also indicates that adolescents who perpetrate overt forms of bullying (e.g., physical aggression and property destruction) have lower levels of fear reactivity and effortful control and commit more direct aggressive acts that generally break social norms (28), compared to perpetrators of covert violence (60).

Understanding how food consumption could be associated with bullying perpetration (e.g., via deviant behaviors) is essential for the adoption of approaches that can prevent or mitigate violent behaviors among adolescents since unhealthy eating represents a modifiable risk factor that can be improved through nutrition promotion initiatives (57, 61) which incorporate healthy dietary components (e.g., vegetable and fruit consumption) into the adolescents’ diet (62). Some studies suggest incorporating these dietary components during the earliest stages of the life course (63, 64), as they may help to reduce early-onset deviant behaviors and, in turn, decrease the perpetration of bullying. However, nutritional interventions during later stages of the life course can also generate beneficial effects (19, 65), especially in schools, which can encourage the adoption of healthy behaviors (e.g., healthy diets, physical activity, rather than substance use, and delinquency) that improve adolescents’ quality of life (66). In Brazil, specifically in public schools, the National School Feeding Program (*Programa Nacional de Alimentação Escolar* – PNAE) encourages and ensures the right to adequate and healthy food for children and adolescents (67).

In schools, the literature indicates that teachers may influence the way students interact with each other (68), guiding and shaping their behavior through classroom pedagogical strategies (69–71) such as circle time (or “group thinking time”). In this activity, students sit in a circle and the teacher encourages them to explore issues relevant to the class (e.g., respecting the rights of others, bullying) (66). Thus, the adolescents acquire school connectedness (72) and internalize the moral norms of a safer and equal school environment, manifesting less aggressive and delinquent behaviors (73, 74). The partnership between teachers, school adolescents, and family is very important for young people to understand moral norms about deviant behavior and bullying and what is right and wrong in peer relationships (75, 76).

To the best of our knowledge, this is the first study that assessed the association between an ultra-processed dietary pattern and different types of bullying, valuing its intentionality and repetitive features (77), and the mediating role of deviant behaviors on this association. This analysis allowed the obtainment of different mediation percentages depending on the type of perpetration reported by a significant sample of adolescents (overall response rate: 94.8%).

The SP-PROSO questions about food consumption in the past 7 days are similar to questions from the previously validated PeNSE questionnaire (Brazilian National School-Based Health Survey) in Brazil (78), allowing a broad assessment of food consumption even considering a small number of healthy and ultra-processed foods in the dietary patterns. A better evaluation of adolescent’s eating habits was possible, through a combination of different food items and nutrients (79). Moreover, following recommendations for culturally sensitive translations (80, 81), the instrument was translated into Portuguese and pre-tested in five schools to better assess the phrasing and understanding of the questions by the students (30).

Despite the strengths, some limitations in our study warrant consideration. First, the study’s cross-sectional design does not allow us to establish causal inferences between the ultra-processed dietary pattern and types of bullying perpetration. Future longitudinal studies should explore the possible causal links between these variables. Second, considering that SP-PROSO is a school-based survey, only adolescents attending school and present in class on during data collection moments were included, which means that our results may not apply to perpetrators of bullying who miss classes or are not enrolled in regular education (82). There was a notable sample loss, but a representative number of Brazilian 9th grade students was still assessed.

Our data were self-reported, which could result in some degree of misreporting (non-differential information bias) in questions about bullying engagement (83) and consequently underestimate the prevalence of this violence. Nonetheless, scores on the 10-item self-report measure of adolescent bullying (Z-PROSO) were validated and provided a reasonable general measure of perpetration that can be used for adolescents of all age groups (84). Other studies have used similar questions for the same exposure (85, 86) and outcomes (87), suggesting that we have properly identified them and enabling comparisons with our results.

We conclude that the association between ultra-processed dietary pattern and bullying may occur via deviant behaviors (mediating effect), with different mediation percentages for each type of bullying perpetration. For physical aggression and property destruction, such association can also happen through pathways other than the mediator tested in this study. These findings are important for the planning and implementation of actions to prevent and manage delinquent behaviors and the practice of school violence, as well as actions to encourage healthy eating among adolescents.

## Data availability statement

The original contributions presented in the study are included in the article/supplementary material, further inquiries can be directed to the corresponding author.

## Ethics statement

The studies involving humans were approved by Ethics and Research Committee of the University of São Paulo School of Medicine. The studies were conducted in accordance with the local legislation and institutional requirements. Written informed consent for participation in this study was provided by the participants’ legal guardians/next of kin.

## Author contributions

LO: Writing – review & editing, Writing – original draft, Visualization, Validation, Resources, Project administration, Methodology, Investigation, Formal analysis, Data curation, Conceptualization. EM: Writing – review & editing, Visualization, Validation, Methodology, Investigation, Formal analysis. RL: Writing – review & editing, Visualization, Validation, Methodology, Investigation, Formal analysis. VG: Writing – review & editing, Visualization, Validation, Methodology, Investigation, Formal analysis. MP: Writing – review & editing, Visualization, Validation, Methodology, Investigation, Funding acquisition, Formal analysis, Data curation. CA: Writing – review & editing, Visualization, Validation, Supervision, Methodology, Investigation, Funding acquisition, Formal analysis, Data curation, Conceptualization.

## References

[1] Akanni OO Olashore AA Osasona SO Uwadiae E. Predictors of bullying reported by perpetrators in a sample of senior school students in Benin City, Nigeria. S Afr J Psychiatry. (2020) 26:a1359. doi: 10.4102/sajpsychiatry.v26i0.1359, PMID: 32161679 PMC7059440

[2] Krug EG Mercy JA Dahlberg LL Zwi AB. The world report on violence and health. Lancet. (2002) 360:1083–8. doi: 10.1016/S0140-6736(02)11133-0 12384003

[3] Olweus D. School bullying: development and some important challenges. Annu Rev Clin Psychol. (2013) 9:751–80. doi: 10.1146/annurev-clinpsy-050212-185516 23297789

[4] Instituto Brasileiro de Geografia e Estatística. Pesquisa nacional de saúde do escolar: 2019. [Internet]. (2021). Available at: https://biblioteca.ibge.gov.br/visualizacao/livros/liv101852.pdf (Accessed Nov 15, 2023).

[5] Solnick SJ Hemenway D. The “Twinkie defense”: the relationship between carbonated non-diet soft drinks and violence perpetration among Boston high school students. Inj Prev. (2012) 18:259–63. doi: 10.1136/injuryprev-2011-040117, PMID: 22025524

[6] Trapp GS Allen KL Black LJ Ambrosini GL Jacoby P Byrne S. A prospective investigation of dietary patterns and internalizing and externalizing mental health problems in adolescents. Food Sci Nutr. (2016) 4:888–96. doi: 10.1002/fsn3.355, PMID: 27826439 PMC5090653

[7] Bruckauf Z Walsh SD. Adolescents’ multiple and individual risk behaviors: examining the link with excessive sugar consumption across 26 industrialized countries. Soc Sci Med. (2018) 216:133–41. doi: 10.1016/j.socscimed.2018.08.029, PMID: 30269866

[8] Jackson DB. Diet quality and bullying among a cross-national sample of youth. Prev Med. (2017) 105:359–65. doi: 10.1016/j.ypmed.2017.06.033, PMID: 29056315

[9] Zahedi H Kelishadi R Heshmat R Motlagh ME Ranjbar SH Ardalan G. Association between junk food consumption and mental health in a national sample of Iranian children and adolescents: the CASPIAN-IV study. Nutrition. (2014) 30:1391–7. doi: 10.1016/j.nut.2014.04.014, PMID: 25280418

[10] Okada LM Marques ES Levy RB Peres MFT Azeredo CM. Association between dietary patterns and bullying among adolescents in São Paulo-Brazil. Int J Offender Ther Comp Criminol. (2022) 68:299–316. doi: 10.1177/0306624X221095017, PMID: 35535611

[11] Black MM. Effects of vitamin B12 and folate deficiency on brain development in children. Food Nutr Bull. (2008) 29:S126–31. doi: 10.1177/15648265080292s117, PMID: 18709887 PMC3137939

[12] Gómez-Pinilla F. Brain foods: the effects of nutrients on brain function. Nat Rev Neurosci. (2008) 9:568–78. doi: 10.1038/nrn2421, PMID: 18568016 PMC2805706

[13] Schoemaker K Mulder H Deković M Matthys W. Executive functions in preschool children with externalizing behavior problems: a Meta-analysis. J Abnorm Child Psychol. (2012) 41:457–71. doi: 10.1007/s10802-012-9684-x, PMID: 23054130

[14] Monteiro CA Cannon G Levy RB Moubarac J-C Louzada MLC Rauber F. Ultra-processed foods: what they are and how to identify them. Public Health Nutr. (2019) 22:936–41. doi: 10.1017/S1368980018003762, PMID: 30744710 PMC10260459

[15] Louzada MLC Cruz GL Silva KAAN Grassi AGF Andrade GC Rauber F. Consumption of ultra-processed foods in Brazil: distribution and temporal evolution 2008–2018. Rev Saude Publica. (2023) 57:12. doi: 10.11606/s1518-8787.2023057004744 37075395 PMC10118420

[16] Levy RB Andrade GC da Cruz GL Rauber F Louzada MLC Claro RM. Three decades of household food availability according to NOVA – Brazil, 1987–2018. Rev Saude Publica. (2022) 56:75. doi: 10.11606/s1518-8787.2022056004570, PMID: 35946675 PMC9388064

[17] Wikström P-OH Treiber KH. Violence as situational action. Int J Conf Violence. (2009) 3:75–96. doi: 10.4119/ijcv-2794

[18] Beseler L. Effects on behavior and cognition: diet and artificial colors, flavors, and preservatives. Int Pediatr. (1999) 14:41–3.

[19] Gesch CB Hammond SM Hampson SE Eves A Crowder MJ. Influence of supplementary vitamins, minerals and essential fatty acids on the antisocial behaviour of young adult prisoners: randomized, placebo-controlled trial. Br J Psychiatry. (2002) 181:22–8. doi: 10.1192/bjp.181.1.22, PMID: 12091259

[20] Henggeler SW Sheidow AJ. Empirically supported family-based treatments for conduct disorder and delinquency in adolescents. J Marital Fam Ther. (2012) 38:30–58. doi: 10.1111/j.1752-0606.2011.00244.x, PMID: 22283380 PMC3270911

[21] Loeber R Wung P Keenan K Giroux B Southamer-Loeber M van Kammen WB. Developmental pathways in disruptive child behavior. Dev Psychopathol. (1993) 5:103–33. doi: 10.1017/S0954579400004296

[22] Murray J Farrington DP. Risk factors for conduct disorder and delinquency: key findings from longitudinal studies. Can J Psychiatr. (2010) 55:633–42. doi: 10.1177/070674371005501003, PMID: 20964942

[23] Benton D. The impact of diet on anti-social, violent and criminal behavior. Neurosci Biobehav Rev. (2007) 31:752–74. doi: 10.1016/j.neubiorev.2007.02.002, PMID: 17433442

[24] Oddy WH Robinson M Ambrosini GL O’Sullivan TA de Klerk NH Beilin LJ. The association between dietary patterns and mental health in early adolescence. Prev Med. (2009) 49:39–44. doi: 10.1016/j.ypmed.2009.05.009 19467256

[25] Gonzalvo GO. Delinquent adolescents: health problems and health care guidelines for juvenile correctional facilities. An Esp Pediatr. (2002) 57:345–53. PMID: 12392669

[26] Zupo R Castellana F Sardone R Sila A Giagulli VA Triggiani V. Preliminary trajectories in dietary behaviors during the COVID-19 pandemic: a public health call to action to face obesity. Int J Environ Res Public Health. (2020) 17:7073. doi: 10.3390/ijerph17197073, PMID: 32992623 PMC7579065

[27] De Nucci S Zupo R Castellana F Sila A Triggiani V Lisco G. Public health response to the SARS-CoV-2 pandemic: concern about ultra-processed food consumption. Food Secur. (2022) 11:950. doi: 10.3390/foods11070950, PMID: 35407037 PMC8997472

[28] Aslund C Starrin B Leppert J Nilsson KW. Social status and shaming experiences related to adolescent overt aggression at school. Aggress Behav. (2009) 35:1–13. doi: 10.1002/ab.20286, PMID: 18925634

[29] Newby PK Tucker KL. Empirically derived eating patterns using factor or cluster analysis: a review. Nutr Rev. (2004) 62:177–203. doi: 10.1111/j.1753-4887.2004.tb00040.x 15212319

[30] Peres MFT Eisner M Loch AP Nascimento A Papa CHG Azeredo CM. Violência, bullying e repercussões na saúde: resultados do projeto São Paulo para o desenvolvimento social de crianças e adolescentes (SP-PROSO). Departamento de Medicina Preventiva/FMUSP [Internet]. (2018). Available at: https://sites.usp.br/sp-proso/wp-content/uploads/sites/526/2019/06/relatorio_sp_proso_26_05_2019.pdf (Accessed Nov 15, 2023).

[31] Azeredo CM Rezende LF Canella DS Claro RM Castro IRR Luiz OC. DietaryintakeofBrazilianadolescents. Public Health Nutr. (2015) 18:1215–24. doi: 10.1017/S1368980014001463, PMID: 25089589 PMC10271650

[32] Costello AB Osborne J. Best practices in exploratory factor analysis: four recommendations for getting the most from your analysis. Pract Assess Res Eval. (2005) 10:7. doi: 10.7275/JYJ1-4868

[33] Hutcheson GD Sofroniou N. The multivariate social scientist: Introductory statistics using generalized linear models SAGE (1999).

[34] Trajtenberg N Eisner M. Towards a more effective violence prevention policy in Uruguay. Institute of Criminology University of Cambridge (2015) isbn:978-9974-8470-6-4.

[35] Nivette A Echelmeyer L Weerman F Eisner M Ribeaud D. Understanding changes in violent extremist attitudes during the transition to early adulthood. J Quant Criminol. (2022) 38:949–78. doi: 10.1007/s10940-021-09522-9, PMID: 36340926 PMC9626430

[36] Sweeten G. Scaling criminal offending. J Quant Criminol. (2012) 28:533–57. doi: 10.1007/s10940-011-9160-8

[37] Nivette A Trajtenberg N Eisner M Ribeaud D Peres MFT. Assessing the measurement invariance and antecedents of legal cynicism in São Paulo, Zurich, and Montevideo. J Adolesc. (2020) 83:83–94. doi: 10.1016/j.adolescence.2020.06.007 32763619

[38] Brauer JR Tittle CR. When crime is not an option: inspecting the moral filtering of criminal action alternatives. Justice Q. (2017) 34:818–46. doi: 10.1080/07418825.2016.1226937

[39] Emmert AD Carlock AL Lizotte AJ Krohn MD. Predicting adult under- and over-reporting of self-reported arrests from discrepancies in adolescent self-reports of arrests: a research note. Crime Delinq. (2017) 63:412–28. doi: 10.1177/0011128715575141

[40] Piquero AR Schubert CA Brame R. Comparing official and self-report records of offending across gender and race/ethnicity in a longitudinal study of serious youthful offenders. J Res Crime Delinq. (2014) 51:526–56. doi: 10.1177/0022427813520445

[41] Skelly AC Dettori JR Brodt ED. Assessing bias: the importance of considering confounding. Evid Based Spine Care J. (2012) 3:9–12. doi: 10.1055/s-0031-1298595, PMID: 23236300 PMC3503514

[42] DAGitty Online Browser. Available at: http://www.dagitty.net/dags.html# (Accessed Nov 15, 2023).

[43] Greenland S Pearl J Robins JM. Causal diagrams for epidemiologic research. Epidemiology. (1999) 10:37–48. doi: 10.1097/00001648-199901000-00008 9888278

[44] Breen R Karlson KB Holm A. Total, direct, and indirect effects in logit and Probit models. Sociol Methods Res. (2013) 42:164–91. doi: 10.1177/0049124113494572

[45] StataCorp. Stata statistical software: Release 13 StataCorp LP (2013).

[46] Stitt BR. Food & Behavior – nutritional guidelines for correcting behavior. (1999).

[47] Sharma J. Nutritional correlates of aggressive behavior. Violence and conflict resolution. (2009) Contemporary Perspectives, 157–163.

[48] Hibbeln JR Umhau JC Linnoila M George DT Ragan PW Shoaf SE. A replication study of violent and nonviolent subjects: cerebrospinal fluid metabolites of serotonin and dopamine are predicted by plasma essential fatty acids. Biol Psychiatry. (1998) 44:243–9. doi: 10.1016/s0006-3223(98)00143-7, PMID: 9715355

[49] Cintra DE Ropelle ER Moraes JC Pauli JR Morari J Souza CT. Unsaturated fatty acids revert diet-induced hypothalamic inflammation in obesity. PLoS One. (2012) 7:e30571. doi: 10.1371/journal.pone.0030571, PMID: 22279596 PMC3261210

[50] Coll T Eyre E Rodríguez-Calvo R Palomer X Sánchez RM Merlos M. Oleate reverses palmitate-induced insulin resistance and inflammation in skeletal muscle cells. J Biol Chem. (2008) 283:11107–16. doi: 10.1074/jbc.M708700200, PMID: 18281277

[51] Suarez EC Lewis JG Kuhn C. The relation of aggression, hostility, and anger to lipopolysaccharide-stimulated tumor necrosis factor (TNF)-alpha by blood monocytesfrom normal men. Brain Behav Immun. (2002) 16:675–84. doi: 10.1016/S0889-1591(02)00019-3, PMID: 12480498

[52] Kien CL Bunn JY Tompkins CL Dumas JA Crain KI Ebenstein DB. Substituting dietary monounsaturated fat for saturated fat is associated with increased daily physical activity and resting energy expenditure and with changes in mood. Am J Clin Nutr. (2013) 97:689–97. doi: 10.3945/ajcn.112.051730, PMID: 23446891 PMC3607650

[53] Holubcikova J Kolarcik P MadarasovaGeckova A Reijneveld SA van Dijk JP. The mediating effect of daily nervousness and irritability on the relationship between soft drink consumption and aggressive behaviour among adolescents. Int J Public Health. (2015) 60:699–706. doi: 10.1007/s00038-015-0707-6, PMID: 26140860

[54] Tandel KR. Sugar substitutes: health controversy over perceived benefits. J Pharmacol Pharmacother. (2011) 2:236–43. doi: 10.4103/0976-500X.85936, PMID: 22025850 PMC3198517

[55] Kanarek RB. Nutrition and violent behavior. In: Understanding and preventing violence, vol. 2 (1994). 1–33.

[56] Kristjansson AL Sigfusdottir ID Frost SS James JE. Adolescent caffeine consumption and self-reported violence and conduct disorder. J Youth Adolesc. (2013) 42:1053–62. doi: 10.1007/s10964-013-9917-5, PMID: 23358888

[57] Jackson DB Vaughn MG Salas-Wright CP. Poor nutrition and bullying behaviors: a comparison of deviant and non-deviant youth. J Adolesc. (2017) 57:69–73. doi: 10.1016/j.adolescence.2017.03.008, PMID: 28384522

[58] Raine A. The anatomy of violence: the biological roots of crime Vintage (2013).

[59] Du Y. You may be what you eat, can you be violent due to your food? Eur J Biomed Pharm Sci (2019) 6:20–28. ISSN 2349-8870

[60] Terranova AM Morris AS Boxer P. Fear reactivity and effortful control in overt and relational bullying: a six-month longitudinal study. Aggress Behav. (2008) 34:104–15. doi: 10.1002/ab.20232, PMID: 17786968

[61] Wang D Stewart D. The implementation and effectiveness of school-based nutrition promotion programmes using a health-promoting schools approach: a systematic review. Public Health Nutr. (2013) 16:1082–100. doi: 10.1017/S1368980012003497, PMID: 22850118 PMC10271240

[62] Jackson DB. The link between poor quality nutrition and childhood antisocial behavior: a genetically informative analysis. J Crim Justice. (2016) 44:13–20. doi: 10.1016/j.jcrimjus.2015.11.007

[63] Raine A Mellingen K Liu J Venables P Mednick SA. Effects of environmental enrichment at ages 3–5 years on schizotypal personality and antisocial behavior at ages 17 and 23 years. Am J Psychiatry. (2003) 160:1627–35. doi: 10.1176/appi.ajp.160.9.1627, PMID: 12944338

[64] Benton D. The influence of children’s diet on their cognition and behavior. Eur J Nutr. (2008) 47:25–37. doi: 10.1007/s00394-008-3003-x 18683027

[65] Zaalberg A Nijman H Bulten E Stroosma L van der Staak C. Effects of nutritional supplements on aggression, rule-breaking, and psychopathology among young adult prisoners. Aggress Behav. (2009) 36:117–26. doi: 10.1002/ab.20335, PMID: 20014286

[66] World Health Organization. Mental health in schools: A manual. Cairo: WHO Regional Office for the Eastern Mediterranean (2021). Licence: CC BY-NC-SA 3.0 IGO.

[67] Ministry of Education. Resolution no. 6, of May 8, 2020. National Education Development Fund (2020). Available at: https://www.fnde.gov.br/index.php/acesso-a-informacao/institucional/legislacao/item/13511-resolu%C3%A7%C3%A3o-n%C2%BA-6,-de-08-de-maio-de-2020 (Accessed Feb 25, 2023).

[68] Nivette A Obsuth I Ribeaud D Eisner M. Fair teachers, fair police? Assessing the pathways between perceptions of teacher and police Authority in Childhood and Adolescence. J Youth Adolesc. (2022) 51:193–207. doi: 10.1007/s10964-021-01537-6, PMID: 34783955 PMC8828593

[69] Berkowitz MW. What works in values education. Int J Educ Res. (2011) 50:153–8. doi: 10.1016/j.ijer.2011.07.003

[70] Buzzelli CA. The moral implications of teacher-child discourse in early childhood classrooms. Early Child Res Q. (1996) 11:515–34. doi: 10.1016/S0885-2006(96)90020-4

[71] Nucci LP. *Education in the moral domain*. Cambridge: Cambridge University Press (2001).

[72] Loukas A Roalson LA Herrera DE. School connectedness buffers the effects of negative family relations and poor effortful control on early adolescent conduct problems. J Res Adolesc. (2010) 20:13–22. doi: 10.1111/j.1532-7795.2009.00632.x

[73] Obsuth I Murray AL Knoll M Ribeaud D Eisner M. Teacher-student relationships in childhood as a protective factor against adolescent delinquency up to age 17: a propensity score matching approach. Crime Delinq. (2021) 69:727–55. doi: 10.1177/00111287211014153, PMID: 36960348 PMC10026349

[74] Wang C Swearer SM Lembeck P Collins A Berry B. Teachers matter: an examination of student-teacher relationships, attitudes towards bullying, and bullying behavior. J Appl Sch Psychol. (2015) 31:219–38. doi: 10.1080/15377903.2015.1056923

[75] Smetana JG Jambon M Ball C. Normative changes and individual differences in early moral judgments: a constructivist developmental perspective. Hum Dev. (2018) 61:264–80. doi: 10.1159/000492803

[76] Stewart D Sun J Patterson C Lemerle K Hardie M. Promoting and building resilience in primary school communities: evidence from a comprehensive “health promoting school” approach. Int J Ment Health Promot. (2004) 6:26–33. doi: 10.1080/14623730.2004.9721936

[77] Olweus D. Bullying at school. What we know and what we can do Blackwell (1993).

[78] Tavares LF Castro IRR Levy RB Cardoso LO Passos MD Brito FSB. Validade relativa de indicadores de práticas alimentares da Pesquisa Nacional de Saúde do Escolar entre adolescentes do Rio de Janeiro, Brasil. Cad Saúde Pública. (2014) 30:1029–41. doi: 10.1590/0102-311X00000413 24936819

[79] Kant AK. Dietary patterns and health outcomes. J Am Diet Assoc. (2004) 104:615–35. doi: 10.1016/j.jada.2004.01.010 15054348

[80] Behr D Shishido K. The translation of measurement instruments for cross-cultural surveys. In: Wolf C Joye D Smith TW Fu Y, editors. *The SAGE handbook of survey methodology*: SAGE (2016). 269–87.

[81] Eisner M Ribeaud D. Conducting a criminological survey in a culturally diverse context: lessons from the Zurich project on the social development of children. Eur J Criminol. (2007) 4:271–98. doi: 10.1177/1477370807077183

[82] Gubbels J van der Put CE Assink M. Risk factors for school absenteeism and dropout: a meta-analytic review. J Youth Adolesc. (2019) 48:1637–67. doi: 10.1007/s10964-019-01072-5, PMID: 31312979 PMC6732159

[83] Cornell DG Lovegrove PJ Baly MW. Invalid survey response patterns among middle school students. Psychol Assess. (2014) 26:277–87. doi: 10.1037/a0034808, PMID: 24219702

[84] Murray AL Eisner M Ribeaud D Kaiser D McKenzie K Murray G. Validation of a brief self-report measure of adolescent bullying perpetration and victimization. Assessment. (2021) 28:128–40. doi: 10.1177/1073191119858406, PMID: 31280595

[85] Barros MBA Lima MG Azevedo RCS Medina LBP Lopes CS Menezes PR. Depression and health behaviors in Brazilian adults – PNS 2013. Rev Saude Publica. (2017) 51:8s. doi: 10.11606/S1518-8787.2017051007138, PMID: 28591352 PMC5676399

[86] Tavares LF Castro IR Levy RB Cardoso LO Claro RM. Dietary patterns of Brazilian adolescents: results of the Brazilian National School-Based Health Survey (PeNSE). Cad Saude Publica. (2014) 30:2679–90. doi: 10.1590/0102-311X00016814, PMID: 26247996

[87] Murray AL Zych I Ribeaud D Eisner M. Developmental relations between ADHD symptoms and bullying perpetration and victimization in adolescence. Aggress Behav. (2021) 47:58–68. doi: 10.1002/ab.21930, PMID: 32895934

